# Comparative metabolic profiling of *Haberlea rhodopensis, Thellungiella halophyla*, and *Arabidopsis thaliana* exposed to low temperature

**DOI:** 10.3389/fpls.2013.00499

**Published:** 2013-12-11

**Authors:** Maria Benina, Toshihiro Obata, Nikolay Mehterov, Ivan Ivanov, Veselin Petrov, Valentina Toneva, Alisdair R. Fernie, Tsanko S. Gechev

**Affiliations:** ^1^Department of Plant Physiology and Plant Molecular Biology, University of PlovdivPlovdiv, Bulgaria; ^2^Institute of Molecular Biology and BiotechnologyPlovdiv, Bulgaria; ^3^Department Willmitzer, Max Planck Institute of Molecular Plant PhysiologyPotsdam-Golm, Germany; ^4^Department of Molecular Biology, Institute of Biochemistry and Biology, University of PotsdamPotsdam, Germany

**Keywords:** *Arabidopsis thaliana*, *Haberlea rhodopensis*, low temperature stress, metabolite profiling, *Thellungiella halophila*

## Abstract

*Haberlea rhodopensis* is a resurrection species with extreme resistance to drought stress and desiccation but also with ability to withstand low temperatures and freezing stress. In order to identify biochemical strategies which contribute to Haberlea's remarkable stress tolerance, the metabolic reconfiguration of *H. rhodopensis* during low temperature (4°C) and subsequent return to optimal temperatures (21°C) was investigated and compared with that of the stress tolerant *Thellungiella halophyla* and the stress sensitive *Arabidopsis thaliana*. Metabolic analysis by GC-MS revealed intrinsic differences in the metabolite levels of the three species even at 21°C. *H. rhodopensis* had significantly more raffinose, melibiose, trehalose, rhamnose, *myo*-inositol, sorbitol, galactinol, erythronate, threonate, 2-oxoglutarate, citrate, and glycerol than the other two species. *A. thaliana* had the highest levels of putrescine and fumarate, while *T. halophila* had much higher levels of several amino acids, including alanine, asparagine, beta-alanine, histidine, isoleucine, phenylalanine, serine, threonine, and valine. In addition, the three species responded differently to the low temperature treatment and the subsequent recovery, especially with regard to the sugar metabolism. Chilling induced accumulation of maltose in *H. rhodopensis* and raffinose in *A. thaliana* but the raffinose levels in low temperature exposed Arabidopsis were still much lower than these in unstressed Haberlea. While all species accumulated sucrose during chilling, that accumulation was transient in *H. rhodopensis* and *A. thaliana* but sustained in *T. halophila* after the return to optimal temperature. Thus, Haberlea's metabolome appeared primed for chilling stress but the low temperature acclimation induced additional stress-protective mechanisms. A diverse array of sugars, organic acids, and polyols constitute Haberlea's main metabolic defence mechanisms against chilling, while accumulation of amino acids and amino acid derivatives contribute to the low temperature acclimation in Arabidopsis and Thellungiella. Collectively, these results show inherent differences in the metabolomes under the ambient temperature and the strategies to respond to low temperature in the three species.

## Introduction

The small but diverse group of resurrection plants exhibit a remarkable adaptation to extreme drought stress. In absence of water supply, they can tolerate desiccation of their vegetative tissues to air dried state and quickly regain normal appearance and metabolism upon rehydration (Dinakar et al., 2012; Gechev et al., 2012). *Haberlea rhodopensis* is a desiccation-tolerant species, perennial herbaceous plant endemic to several mountains in the Balkan Peninsula in South-Eastern Europe (Gechev et al., 2013a). It is also an ancient plant, a glacial relic, which might have acquired its defence mechanisms a long time ago. As it is exposed to the harsh winter conditions and subzero temperatures in these latitudes, this species additionally evolved mechanisms to withstand chilling and freezing stress.

Earlier studies on resurrection plants indicated that complex and diverse mechanisms can contribute to their desiccation tolerance. These include alterations of sugar metabolism, reconfiguration of the cell wall, inhibition of growth and photosynthesis, rapid induction of late embryogenesis abundant (LEA) and small heat shock proteins, accumulation of phenolic antioxidants, upregulation of antioxidant enzymes, aldehyde dehydrogenases, and other protective enzymes (Kirch et al., 2001; Mowla et al., 2002; Battaglia et al., 2008; Rodriguez et al., 2010; Van Den Dries et al., 2011; Moore et al., 2012; Gechev et al., 2013a). Transcriptional re-programming and metabolome re-adjustments are important elements of this stress defence strategy (Rodriguez et al., 2010; Oliver et al., 2011; Yobi et al., 2012, 2013; Gechev et al., 2013a). However, little is known about the molecular responses of resurrection species to low temperatures and no resurrection species has been investigated in terms of metabolome reconfiguration during low temperature stress.

Exposure to freezing environments leads to serious damage of the plant cell by ice formation and dysfunction of cellular membranes. Many plant species increase freezing tolerance during exposure to non-freezing low temperature by a process known as “cold acclimation.” The molecular basis of this process has been extensively studied in *Arabidopsis thaliana*, which is considered sensitive to cold stress, and the contribution of particular metabolites including compatible solutes and the transcriptional regulatory network has been elucidated. For instance, accumulation of sugars is considered to play an important role in cold acclimation (Hannah et al., 2006) and transcription factors such as CBF3/DREB1A play a central role to control this process (Cook et al., 2004). *Thellungiella halophyla* is a close relative of *A*. *thaliana* that has been suggested to possess the characteristics of an extremophile, i.e., high tolerance to salinity, freezing, nitrogen-deficiency, and drought stress (Lee et al., 2012). For this reason, Thellungiella has been analyzed in comparison to Arabidopsis to elucidate the mechanisms that confer tolerance against abiotic stress. Although some accessions of Thellungiella are not extremophile with regard to freezing tolerance, others, including Yukon, show significantly higher tolerance than any *Arabidopsis* accessions (Lee et al., 2012). The metabolite profiling data show different metabolic adaptation strategies between these two species (Lee et al., 2012), indicating specific cold acclimation processes which lead to the different levels of cold tolerance. Recent studies on *Picea sitchensis* and *Fragaria vesca* confirmed the notion that specific cold acclimation processes exist (Dauwe et al., 2012; Rohloff et al., 2012). The desiccation tolerance of *H. rhodopensis* outperforms both Thellungiella and Arabidopsis. Furthermore, Haberlea can withstand freezing temperatures, suggesting distinctive cold acclimation strategies allowing high freezing tolerance in this species. The main aim of this study was to reveal the metabolic changes of *H. rhodopensis* during low temperature treatment and subsequent return to optimal growth temperature. Comparison of the strategies for metabolic adaptation to cold in *H*. *rhodopensis*, *T*. *halophila* and *A*. *thaliana* as representatives of resurrection plants, extremophiles and non-extremophiles, respectively, was carried out to highlight the differences and the common pathways these species employ to adapt to low temperatures. The results suggest the importance of metabolite composition under non-stress conditions as a pre-adaptation strategy and point out the diverse low-temperature stress responses in these three species which likely contribute to the different levels of stress tolerance.

## Materials and methods

### Plant material, growth conditions, and low temperature treatment

*A. thaliana* ecotype Col-0 was obtained from the Nottingham Arabidopsis Stock Centre (NASC, http://arabidopsis.info/); *H. rhodopensis* was initially collected from the Rhodope mountains and subsequently maintained in a climate-controlled room on soil taken from its natural habitat as described (Gechev et al., 2013a,b); *T. halophila* ecotype Yukon was obtained from Dr. Yang-Ping Lee and Dr. Dirk Hintcha, Max-Planck Institute of Molecular Plant Physiology, Potsdam-Golm, Germany.

Plants were grown in a climate room on soil at 21°C, 40 μmol m^−2^ s^−1^ light intensity, 16/8 light/dark photoperiod, and relative humidity 70%. Rosette leaves from all three species were used as samples. Low temperature stress was applied by placing the plants in a plant growth chamber at 4°C, 40 μmol m^−2^ s^−1^ light intensity, 16/8 light/dark photoperiod, and relative humidity 70%. Samples were taken after 3 days of chilling. Subsequently, plants were transferred back to the normal growth conditions (21°C, 40 μmol. m^−2^ s^−1^ light intensity, 16/8 light/dark photoperiod, and relative humidity 70%), and samples were taken after 3 days of recovery. The duration of the low temperature treatment and the recovery period were chosen because longer stress periods interfered with the development of Arabidopsis and are supposed to induce secondary effects on the metabolite profile. In all cases, plant material was immediately frozen in liquid nitrogen, ground into fine powder and stored at −80^°^C until further analysis. Six biological replicates were used for the analyses.

### Measurements of anthocyanins, malondialdehyde, and reduced and oxidized glutathione

The anthocyanins and malondialdehyde were measured photometrically as described by Gechev et al. (2013a,b). Briefly, anthocyanins were extracted with 1% HCl in methanol and the anthocyanin content was determined by reading the absorbance at 530 nm. Correction for non-specific absorption of photosynthetic pigments was done at 657 nm and the final anthocyanin amount, calculated as A_530_ − 0.25 A_657_, was normalized by the fresh weight of the samples. Malondialdehyde was extracted using 1 ml 0.25% thiobarbituric acid dissolved in 10% trichloroacetic acid. After heating the extracts at 85°C for 30 min and rapid chilling on ice, the pellets were removed by centrifugation and the specific absorbance read at 532 nm (the peak of malondialdehyde-thiobarbituric acid complex). Correction for non-specific absorbance at 600 nm was made, malondialdehyde concentration calculated using an extinction coefficient ε_532_ 155 mM^−1^ cm^−1^, and values normalized by the fresh weight of the samples.

Glutathione, total and oxidized, was measured by an enzymatic assay essentially as described by Mehterov et al. (2012). The method relies on the GR-dependent reduction of DTNB, monitored at 412 nm. Briefly, samples were homogenized in 1 ml 5% sulfosalicylic acid (Sigma-Aldrich, St. Louis, Missouri, USA) made in 0.1 M potassium phosphate buffer (pH 7.6/5 mM EDTA) on ice. Aliquots of neutralized extract were mixed with 1.2 mM DNTB (Sigma-Aldrich, St. Louis, Missouri, USA) and 0.3 mM NADPH in 0.1 M potassium phosphate buffer (pH 7.6/5 mM EDTA) and the reaction was started by the addition of 1 U glutathione reductase (Sigma-Aldrich, St. Louis, Missouri, USA). The increase in A_412_ was monitored for 1 min. Oxidized glutathione was measured by the same principle after incubation of neutralized extract with 2 μl 2-vinylpyridine (Sigma-Aldrich, St. Louis, Missouri, USA) for 1 h at room temperature to complex the reduced glutathione. To remove excess 2-vinylpyridine, the derivatized solution was treated with diethyl ether. Reduced glutathione was determined as the difference between total and oxidized glutathione.

### RNA isolation and quantitative RT-PCR

RNA from frozen leaf material was extracted with Trizol Reagent (Invitrogen, Life Technologies, Carlsbad, California, USA) according to the manufacturer's recommendations. Ten micrograms of total RNA was treated with DNA-free™ Kit (Ambion) to remove any DNA contamination. RNA integrity was checked on 1% (w/v) agarose gel and concentration measured with a Nanodrop ND-2000 Spectrophotometer before and after DNAse I digestion. Additionally, the quality and integrity of the RNA samples were analyzed on an RNA 6000 Lab-on-a-Chip using the Bioanalyzer 2100 (Agilent Technologies, Santa Clara, CA, USA). cDNA was synthesized from 2 μg of total RNA using RevertAid™ First Strand cDNA Synthesis Kit (Fermentas, Thermo Fisher Scientific, Waltham, Massachusetts, USA) with oligo-dT primers, according to the manufacturer's instructions.

For qRT-PCR analysis, *A. thaliana* and *T. halophila* chilling- and drought stress-responsive genes were selected based on literature data and information from colleagues (Carvallo et al., 2011; Zou et al., 2011; Y. Ping Lee, personal communication). These included genes encoding two drought stress transcription factors DREB2A and DREB2B in Arabidopsis and DREB2B in Thellungiella, genes encoding LEA proteins in both species, the cold stress-inducible *COR15A, COR47, RD29A* genes in Arabidopsis, and *ELIP2, ADH1*, and *RD29B* genes in Thellungiella. Some of these genes, such as *RD29A/RD29B* (responsive to dehydration), are reported to respond to dehydration as well as to low temperature stress, due to the cellular dehydration that occurs at low temperatures, while others are believed to be more stress-specific. As there were no studies on the molecular responses to low temperatures in Haberlea, genes for the transcriptional analysis were chosen based on our previous analysis during dehydration. The genes were selected from those most regulated by dehydration (Gechev et al., 2013a) and homologs of cold-inducible genes in other species including the temperature-induced lipocalin and the cold-induced glucosyl transferase. The genes of Arabidopsis and Thellungiella are very close to each other in terms of sequence homology, as expected for closely related species. The Haberlea's genes are very divergent from the other two species but nevertheless serve as useful stress markers.

Quantitative real-time PCR (qRT-PCR) analysis was performed using an ABI PRISM 7900 HT PCR instrument (Applied Biosystems, Life Technologies, Carlsbad, California, USA). Primers for the qRT-PCR analysis were designed using the Primer3 software. The genes and corresponding primer pairs from the three species used for the analysis are listed in Supplemental Table 1. All reactions contained 10 μL of SYBR Green Master Mix (Applied Biosystems, Life Technologies, Carlsbad, California, USA), 25 ng of cDNA, and 200 nM of each gene-specific primer in a final volume of 20 μL. The qRT-PCR reactions were executed using the following program: 50°C for 2 min, 95°C for 10 min, followed by 40 cycles of 95°C for 15 s and 60°C for 1 min.

Relative mRNA abundance was calculated using the comparative 2^−Δ Δ^Ct method and normalized to the corresponding reference gene levels (Schmittgen and Livak, 2008). Fold changes in gene expression were calculated and the resulting data sets were log transformed and visualized by Multi Experiment Viewer (MEV)-created heat maps. Two biological and two technical repetitions were performed for each gene.

### Metabolite profiling

Profiling of primary metabolites was conducted using an established gas chromatography mass spectrometry (GC-MS) protocol exactly as described by Lisec et al. (2006). The metabolites were extracted from 100 mg frozen material by methanol and polar metabolites were isolated by phase separation using chloroform. The polar phase was taken and aliquots of 150 μl were dried for further analysis. The metabolites were derivatized with methoxyamine-HCl and *N*-Methyl-*N*-(trimethylsilyl)trifluoroacetamide and subjected to GC-MS analysis. Samples were analyzed by a Gas chromatograph, 6890N (Agilent Technologies, Santa Clara, CA) connected to Pegasus III time-of-flight mass spectrometer (Leco Instruments, St. Joseph, MI) using a MDN-35 capillary column (Macherey-Nagel, Düren, Germany). Chromatograms and mass spectra were evaluated by Chroma TOF® 4.2 (Leco, St Joseph, MI) and TagFinder 4.0 (Luedemann et al., 2008) for the quantification and annotation of the peaks using the MPI Golm Metabolome Database (GMD, http://gmd.mpimp-golm.mpg.de/, Kopka et al., 2005). The parameters used for the identification of the metabolite are summarized following the way recommended in Fernie et al. (2011) as Supplemental Table 3. The amount of metabolites was analyzed as relative metabolite abundance calculated by normalization of signal intensity to that of ribitol which was added as an internal standard and then by the fresh weight of the material. The whole dataset is provided in Supplemental Table 4. Metaboanalyst (www.metaboanalyst.ca, Xia et al., 2012) was used for data analysis including principal component analysis (PCA).

## Results

### Characterization of the physiological responses of *H. rhodopensis, T. halophyla*, and *A. thaliana* to chilling treatment and subsequent recovery

**Figure 1:**
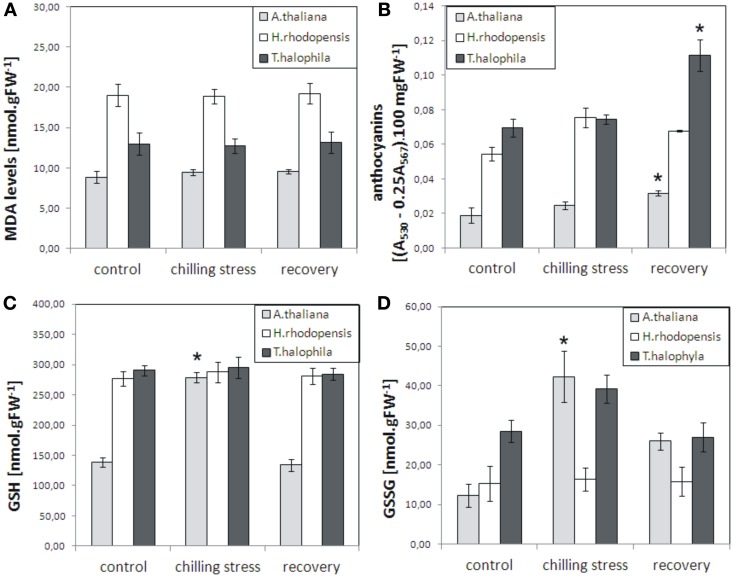
**Malondialdehyde (MDA), anthocyanins, reduced glutathione (GSH), and oxidized glutathione (GSSG) levels in *A. thaliana, H. rhodopensis*, and *T. halophila* exposed to low temperature stress and subsequent recovery**. Plants grown at optimal conditions (controls) at 21°C, 40 μmol. m^−2^ s^−2^ light intensity, 16/8 light/dark photoperiod, and relative humidity 70%, were subjected to chilling (4°C) for 3 days and then returned to 21°C for 3 days for recovery. **(A)**, MDA content; **(B)**, anthocyanins; **(C)**, GSH levels; **(D)**, GSSG levels. MDA, GSH, and GSSG are expressed as nmol per gram fresh weight (FW), while anthocyanins are presented as the difference in absorbencies between 530 and 567 nm (A_530_ − 0.25 A_567_) per 100 mg fresh weight. Asterisks indicate significant difference between controls and low temperature-treated or recovered plants (*P* ≤ 0.05, student's *t*-test). The data are means ± s.e.m. of three replicates.

To evaluate the influence of low temperatures on the three species, plants were inspected for any visible damage and a number of physiological parameters were measured: malondialdehyde levels, which are indicators of lipid peroxidation and oxidative stress; chlorophyll pigments, which normally decrease during severe stress; reduced and oxidized glutathione, which increase as a result of various stresses and their ratio indicates the redox status of the cell. Exposure of *H. rhodopensis, T. halophyla*, and *A. thaliana* to 4°C for 3 days did not cause any visible tissue damage or cell death (data not shown), nor any significant increase in lipid peroxidation as judged by determination of malondialdehyde levels (Figure 1). Furthermore, no visible decrease in turgor or wilting was observed. In control conditions, Haberlea and Thellungiella had 2-fold higher levels of glutathione than Arabidopsis (Figure 1). While the reduced (GSH) and oxidized (GSSG) glutathione remained unchanged during cold treatment and recovery in Haberlea and Thellungiella, both GSH and GSSH increased significantly in Arabidopsis during cold. In Arabidopsis, GSH then returned to initial values on recovery, while GSSH decreased but was still higher than in unstressed controls (Figure 1). Unstressed Haberlea and Thellungiella also displayed 3- and 4-fold higher levels of anthocyanins than Arabidopsis, respectively (Figure 1). In Arabidopsis and Thellungiella, anthocyanins increased on recovery (Figure 1).

### Low temperature- and dehydration-responsive marker genes are induced by chilling treatment in *H. rhodopensis, A. thaliana*, and *T. halophyla*

**Figure 2:**
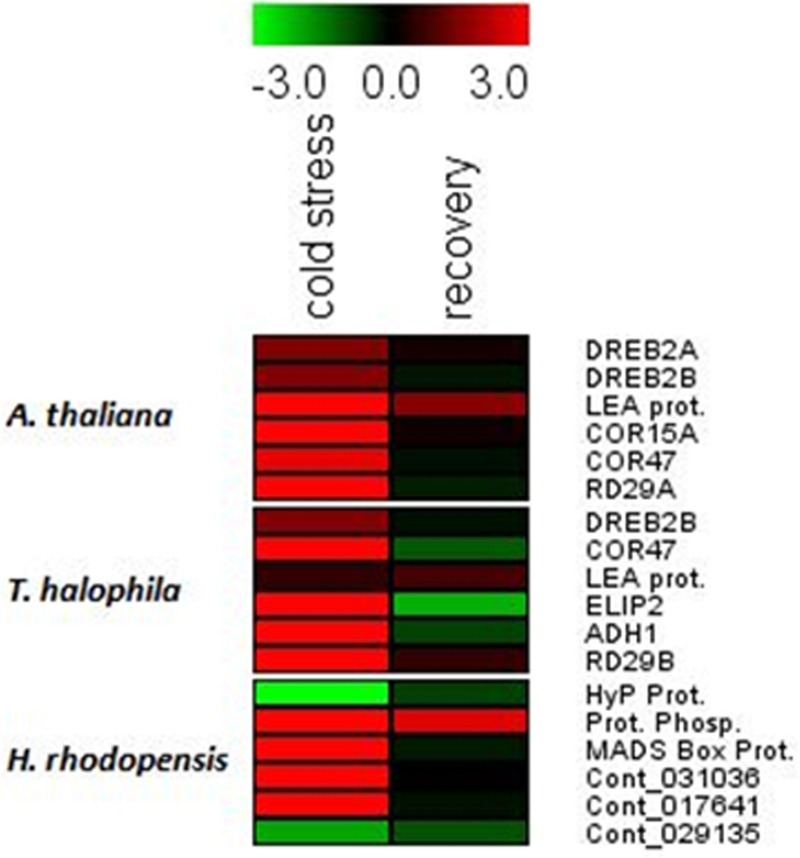
**Drought- and cold-responsive genes are induced by the low temperature treatment in *A. thaliana, H. rhodopensis*, and *T. halophila***. Plants grown at optimal conditions at 21°C, 40 μmol. m^−2^ s^−2^ light intensity, 16/8 light/dark photoperiod, and relative humidity 70%, were subjected to chilling (4°C) for 3 days and then returned to 21°C for recovery. Fully hydrated plants grown at 21°C were used as a reference point for the qRT-PCT analysis. Red colors and positive values depict induced genes while green colors and negative values depict repressed genes. The data are log ratios, average means of two replicates. The genes and primer combinations used are given in Supplemental Table 1, while the exact values of induction/repression are given in Supplemental Table 2.

Cold treatment of *H. rhodopensis, T. halophyla*, and *A. thaliana* resulted in induction of low temperature- and dehydration-responsive marker genes in all three species (Figure 2, Supplemental Table 2). The data shows that cold treatment at 4°C for 3 days induced significantly the *COR15A, COR47*, and *RD29A* genes of Arabidopsis and *RD29B* of Thellungiella, as well as the LEA gene of Arabidopsis and the *ELIP2* and *ADH1* genes of Thellungiella (Figure 2, Supplemental Table 2). The *DREB2A* and *DREB2B* genes of Arabidopsis were not upregulated, indicating that the low temperature treatment did not cause dehydration. In Haberlea, four of the six selected genes were induced by the low temperature, including the temperature-induced lipocalin and the cold-induced glucosyl transferase. A protein phosphatase gene had the highest level of induction; interestingly, the same gene showed the strongest induction by dehydration (Gechev et al., 2013a). With the exception of this protein phosphatase, the levels of all Arabidopsis, Thellungiella, and Haberlea genes returned to normal values upon recovery from chilling (Figure 2). Taken together, these results indicate all three species induced cold response by the low-temperature treatment and recovered following the treatment.

### Metabolite profiles of *H. rhodopensis, A. thaliana*, and *T. halophyla* under non-stress conditions and their reconfigurations during low temperature and subsequent recovery

**Figure 3:**
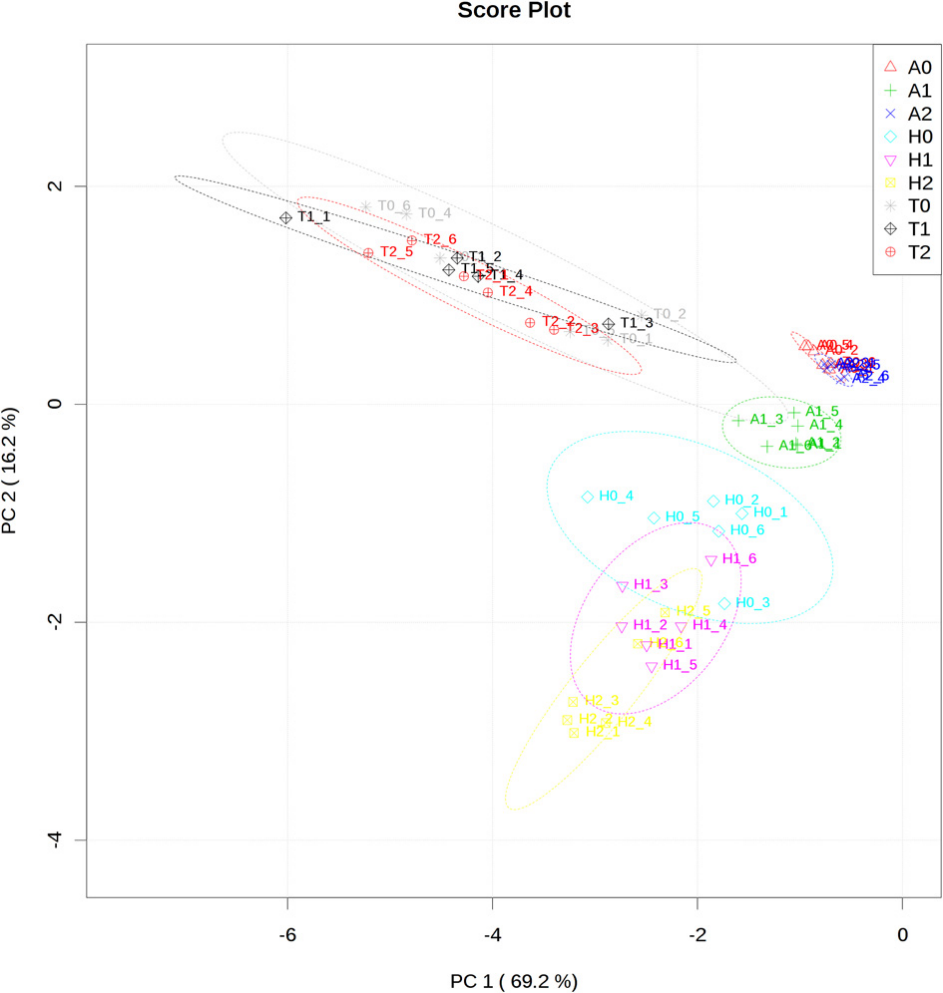
**Principal component analysis of metabolite profiles of leaves from *A. thaliana* (A), *T. halophyla* (T) and *H. rhodopensis* (H) under optimal growth condition (0) and following cold treatment (1) and subsequent recovery (2)**. Each point represents an individual biological replicate. Plotting of the first and second component is shown. The circles indicate the 95% confident regions.

**Figure 4:**
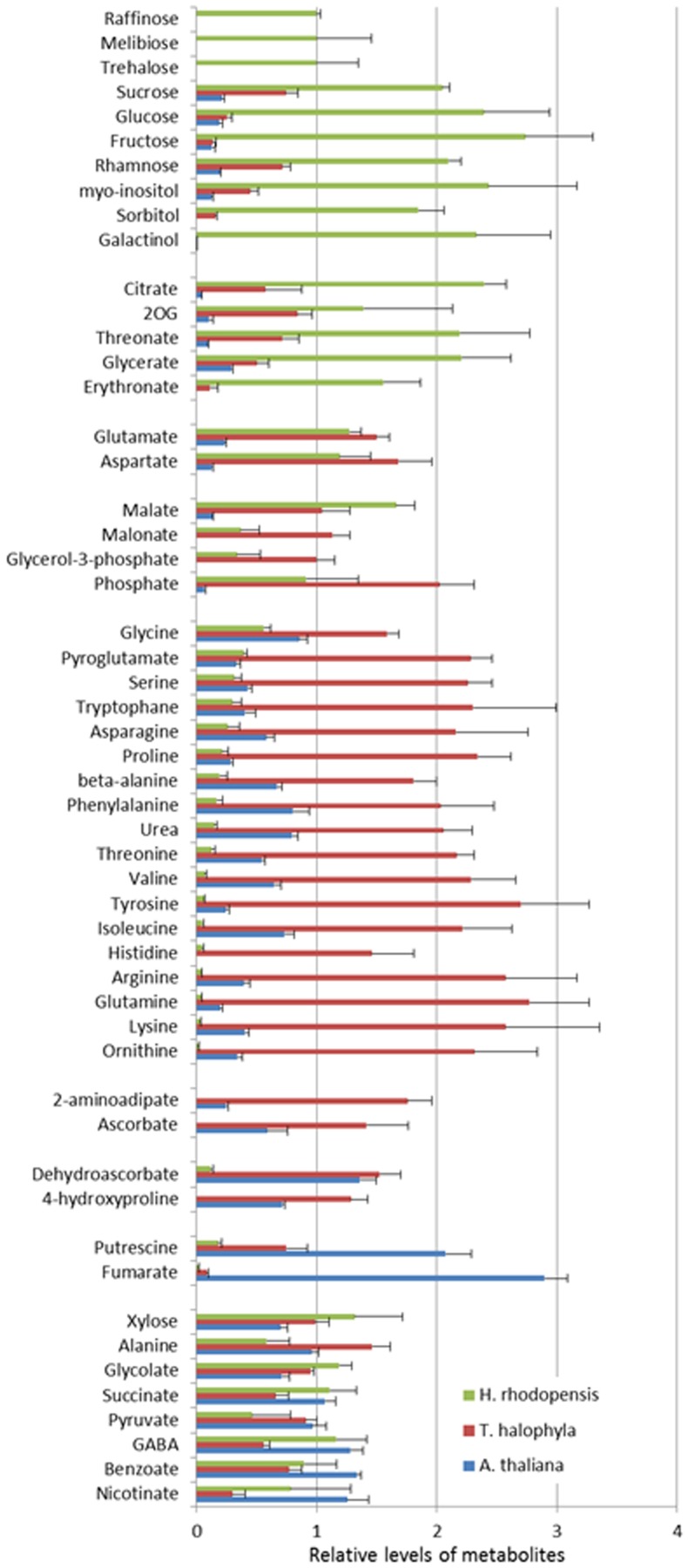
**Comparison of metabolite levels in *A. thaliana* (blue), *T. halophyla* (red) and *H. rhodopensis* (green) under pre-stress condition**. Metabolite levels are normalized by the means of all samples and presented as mean + s.e.m. of six biological replicates.

The measurement of the relative metabolite levels in *H. rhodopensis* and subsequent comparison with *A. thaliana* and *T. halophyla* showed substantial differences between the three species. PCA revealed the differences in the metabolite profiles of the three species in normal growth condition rather than the changes during cold treatment in the same species (Figure 3). *A*. *thaliana* displayed global changes in metabolite levels during the cold treatment while the other two species did not (Figure 3). The metabolite levels under control conditions were compared in Figure 4. *A. thaliana* had much higher levels of putrescine and fumarate than the other two species. Unique features of the Haberlea metabolome were the high level of many sugars and sugar alcohols including glucose, fructose, sucrose, trehalose, rhamnose, raffinose, galactinol, myo-inositol, and sorbitol (Figure 4). This plant also accumulated some organic acids including two TCA cycle intermediates, citrate, and 2-oxoglutarate. Thellungiella, on the other hand, had the highest levels of amino acids including arginine, asparagine, threonine, pyroglutamate, histidine, phenylalanine, valine, glutamine, lysine, isoleucine, ornithine, tyrosine, beta-alanine, serine, tryptophane, and proline (Figure 4). Another observation was the lower levels of ascorbate and dehydroascorbate in Haberlea, than in Arabidopsis and Thellungiella (Figure 4).

**Figure 5:**
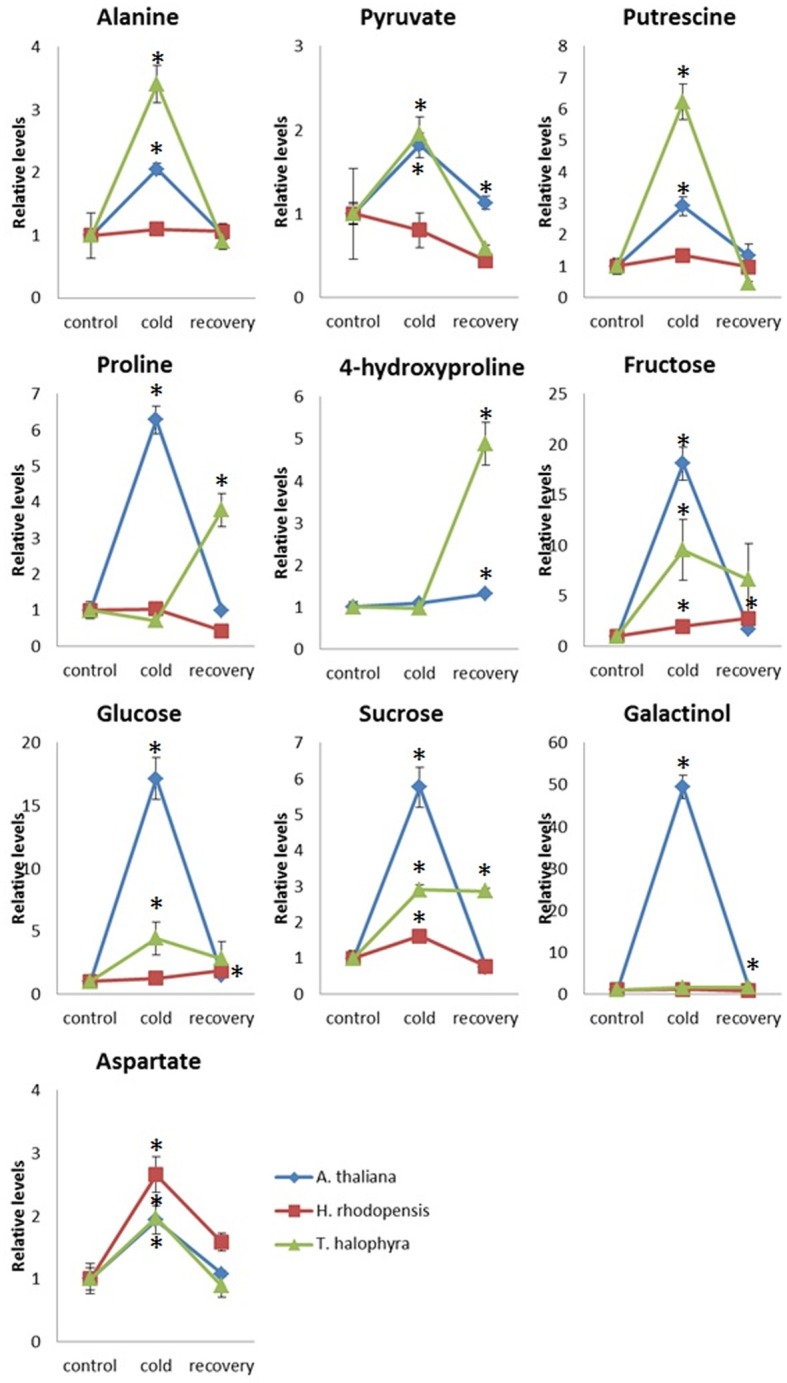
**Changes of metabolite levels during cold treatment and subsequent recovery**. The levels of metabolites were normalized by those at control conditions in each plant species. Ten metabolites which showed statistically significant changes following the treatment are shown. The values are presented as means ± s.e.m. of six biological replicates. Asterisks indicate significant differences by student's *t*-test (*P* < 0.05) of the treated samples compared to the control time points in each species. Blue diamond, *A. thaliana*; red square, *H. rhodopensis*; green triangle, *T. halophyla*.

In addition to these intrinsic metabolic differences, the three species showed distinct metabolic responses to the low temperature treatment. All of them accumulated sucrose, fructose and glucose during cold treatment indicating that accumulation of these sugars is a common response in cold acclimation. Raffinose was detected only after cold treatment in Arabidopsis and Thellungiella, while it was abundant already under normal conditions in Haberlea (Supplemental Table 4). Whilst maltose was not detectable in Arabidopsis, it was clearly detectable in Thellungiella and Haberlea samples following cold treatment and recovery, where it accumulated to much higher levels (Supplemental Table 4). In addition, aspartate transiently accumulated under cold treatment in all three species although the relevance of this to cold acclimation is currently unclear. As suggested by PCA analysis (Figure 3), Arabidopsis transiently accumulated many metabolites including galactinol and proline (Figure 5). An increase in alanine, putrescine and pyruvate was observed both in Arabidopsis and Thellungiella (Figure 5). In many cases, the metabolite changes measured during low temperature treatment were transient. In contrast, there were several examples of sustained accumulation including sucrose, proline, urea and 4-hydroxyproline in Thellungiella (Figure 5).

## Discussion

Despite the numerous studies on resurrection species in the past decade, there is no detailed information on the response of resurrection species to other types of abiotic stresses except drought. In particular, no information is available on the molecular mechanisms of low temperature tolerance in any of the angiosperm resurrection species. We chose to investigate *H*. *rhodopensis*, as this perennial plant can withstand harsh winters in its native habitat and a number of genes associated with low temperature responses are induced by dehydration, suggesting a possible cross-protection (Gechev et al., 2013a). While our main goal was to investigate the metabolic reconfiguration of Haberlea during low temperature treatment and subsequent recovery, comparative analysis with Arabidopsis and Thellungiella highlighted the species-specific metabolic responses as well as the protective mechanisms conserved among evolutionary distant organisms. Due to the differences in morphological and physiological properties of these species, direct comparison of metabolite levels should be done with special care. However, prominent differences (considerered as qualitative) in the metabolic levels and the accumulation pattern during the time course should reflect metabolic feature of the species.

### Low temperature causes cold acclimation responses but no severe oxidative stress in *H. rhodopensis, A. thaliana*, and *T. halophyla*

The duration and magnitude of the low temperature treatment caused neither severe oxidative stress, as judged by the unaltered malondialdehyde and chlorophyll levels, nor cell death in any of the three species. Yet, the low temperatures imposed prominent changes in gene expression and metabolite levels of Haberlea, Arabidopsis, and Thellungiella, indicating that acclimation responses took place in all three species. For Haberlea, these results are original. Although a number of studies investigated different aspects of low temperature responses of Arabidopsis and Thellungiella, no single study covered all aspects of low temperature stress (Cook et al., 2004; Hannah et al., 2006; Alcázar et al., 2011; Carvallo et al., 2011; Lee et al., 2012; Zuther et al., 2012). Furthermore, even small differences between growth conditions or/and experimental set-ups may result in notable differences in metabolite levels, hence the two species were also included in the experiments, instead of retrieving data for them from the literature.

The higher basal levels of anthocyanins in Haberlea and Thellungiella compared with Arabidopsis (3- and 4-fold higher, respectively) may be a part of a pre-adaptation strategy, ensuring a higher level of basal protection, although interpretation of such cross-species comparisons need to be done carefully. Anthocyanins are well-known antioxidants and protectors against oxidative stress. Recently, tomato plants expressing higher levels of anthocyanins have been demonstrated to produce fruits with expended shelf life, delayed ripening, and increased resistance to the fungal pathogen *Botrytis cinerea*, due to the altered spreading of ROS burst after infection (Zhang et al., 2013). Furthermore, the oxidative stress-tolerant Arabidopsis mutant *oxr1* was shown to have much higher levels of anthocyanins than the wild type and much stronger induction of anthocyanin synthesis upon oxidative stress (Gechev et al., 2013b).

The higher levels of the prominent antioxidant and redox regulator glutathione in Haberlea and Thellungiella may also contribute to the higher basal stress tolerance of the two species relative to Arabidopsis. It is interesting to note that the levels of reduced glutathione remain unchanged in Haberlea and Thellungiella during chilling and subsequent recovery while the reduced glutathione increases 2-fold in low temperature treated Arabidopsis to reach the levels of the other two species, but then drops again during recovery. Furthermore, oxidized glutathione levels do not change in Haberlea during chilling and recovery but increased 3-fold when Arabidopsis was subjected to chilling. This collectively implies that the low temperature changes the oxidative status of the more sensitive Arabidopsis and has less effect on the more tolerant Thellungiella and Haberlea.

### Low temperature- and dehydration-responsive marker genes are induced by chilling in *H. rhodopensis, A. thaliana*, and *T. halophyla*

Despite Haberlea's potential in plant biotechnology and biomedical science, the genome of this species remains unsequenced and very limited information is currently available concerning its genes (Apostolova et al., 2012; Georgieva et al., 2012). Recently, the first comprehensive transcriptome and metabolite profiling study of *H. rhodopensis* indicated the types of genes present in Haberlea and how they are expressed during dehydration and rehydration (Gechev et al., 2013a). The low temperature- and drought-responses of Arabidopsis and Thellungiella are well studied (Cook et al., 2004; Hannah et al., 2006; Thomashow, 2010; Carvallo et al., 2011; Zou et al., 2011; Lee et al., 2012), which allowed us to select proper marker genes for use as indicators of stress. The results from this study provide valuable information concerning low temperature-inducible genes that can be used as markers for abiotic stress in Haberlea. The cold-inducible glucosyl transferase (Figure 2, Supplemental Table 2) seems to be specific for low temperature as our expression analysis shows that it is induced under low temperature but not significantly by drought (Gechev et al., 2013a). The other three genes that were upregulated after chilling, those encoding a protein phosphatase, a MADS box protein, and a temperature-induced lipocalin, are also responsive to drought and desiccation and can be used as general markers for these two types of abiotic stress. Further expression studies on these genes during other types of stress will show if they can be utilized as general markers of abiotic stress or they respond only to low temperature- and drought-induced osmotic stress. The protein phosphatase gene is of particular interest, as it was the most strongly induced gene by both low temperature and drought stress (Figure 2; Gechev et al., 2013a).

### Metabolic changes indicated distinctive strategies of cold acclimation in *H. rhodopensis, A. thaliana*, and *T. halophyla*

The three species have very distinct metabolic profiles, clearly visible already in the absence of stress. This suggests the importance of basal metabolic composition for cold tolerance. Metabolic pre-adaptation is considered as a major factor affecting the tolerance to stresses in species and cultivars (Sanchez et al., 2011).

Sugar metabolism in particular plays a major role in several types of abiotic stress responses, especially in tolerance against drought, osmotic stress, and chilling. Haberlea was revealed to possess unique sugar metabolism with a variety of sugars and sugar alcohols of much higher levels than the other two species. Thellungiella, on the other hand, accumulates more sugars and sugar alcohols than Arabidopsis (Gong et al., 2005). Higher expression levels of stress-tolerant genes in Thellungiella and accumulation of several compounds that have protective functions in the presence of osmotic imbalance are evident even under pre-stress conditions (Gong et al., 2005). Recent works in Arabidopsis suggested that it may not be a specific sugar that is important for plant freezing tolerance, but rather that sugars in general may constitute a highly redundant cryoprotective system (Korn et al., 2008; Zuther et al., 2012). The accumulation of a large variety of metabolites is likely to contribute to establishment of a robust system to cope with environmental stresses in Haberlea. Particularly, the higher levels of maltose indicate more intensive starch breakdown. This, together with the much higher levels of the monosaccharides glucose and fructose, appears to fuel sucrose synthesis in Haberlea. In the other two species, the amount of fructose and glucose is much lower, although increases are clearly visible during low temperature conditions. Sucrose, fructose and glucose accumulation seems to be a general response of all the three species to low temperature stress and to osmotic stress in particular (Gechev et al., 2013a). Sucrose accumulation is known as a common response to various environmental stresses (Obata and Fernie, 2012). The much higher levels of sucrose and trehalose in Haberlea than in the other two species imply a role of these two non-reducing disaccharides in both drought and low temperature stress tolerance. They are known to accumulate in resurrection plants during dehydration (Drennan et al., 1993; Ingram and Bartels, 1996; Norwood et al., 2000, 2003; Martinelli, 2008) and can serve as osmoprotectants of biological membranes and can stabilize macromolecular structures (Crowe et al., 1992; Martinelli, 2008). Besides this, both sugars have also signaling properties in lower concentrations. Trehalose and its derivative, trehalose-6-phosphate, are central metabolic regulators in Arabidopsis, influencing carbohydrate status, growth, and energy metabolism (Schluepmann et al., 2003; Lunn et al., 2006; Smeekens et al., 2010). Trehalose-6-phosphate stimulates ADP-glucose pyrophosphorylase, promoting starch synthesis, while trehalose has the opposite effect stimulating starch breakdown (Schluepmann et al., 2003; Smeekens et al., 2010). Since the sugars should be highly accumulated to function as a compatible solute, the qualitatively different levels of trehalose suggests its different roles in the three species, namely as a compatible solute in Haberlea and as a signaling molecule in Arabidopsis and Thellungiella as proposed elsewhere (Carillo et al., 2013).

Haberlea has also much higher levels of raffinose, *myo*-inositol and galactinol, which are precursors of raffinose family oligosaccharides. The higher amount of galactinol observed in Haberlea may be directly linked to protection against abiotic stress. Galactinol and raffinose can protect against oxidative stress, which is a consequence of many abiotic stresses, including chilling (Nishizawa et al., 2008). In Arabidopsis, oxidative stress-mediated induction of galactinol synthases and raffinose synthases is governed by the heat shock transcription factor HsfA2 and eventually leads to elevated levels of galactinol and raffinose (Nishizawa et al., 2008). In a recent study, high levels of galactinol as well as expression of dehydrin and alcohol dehydrogenase genes during cold acclimation correlated with low temperature tolerance in *F. vesca* (Davik et al., 2013). The raffinose family oligosaccharides, including raffinose, stachyose, and verbascose, have been shown to accumulate during osmotic stresses such as drought and chilling and protect from these stresses by water replacement and vitrification (Norwood et al., 2003; Farrant et al., 2007). Indeed, the high levels of raffinose accumulated in Arabidopsis during chilling may be the manner in which this species defends itself from low temperature stress. Haberlea's pathway may also be shifted toward synthesis of the more complex stachyose and verbascose, as it is during drought and desiccation (Gechev et al., 2013a).

Induction of putrescine by the low temperature treatment, observed in Arabidopsis and Thellungiella but not in Haberlea, may be a part of the metabolic reconfigurations of the two species to counteract the stress. Recently, induction of putrescine by cold acclimation was observed in *F. vesca* (Rohloff et al., 2012). They also found cold-induced accumulation of aspartate, which is observed in our case in all three species. Levels of polyamines including putrescine raise under cold exposure in many species. There is considerable evidence for the important roles of polyamine including putrescine in plant defence against cold and other abiotic stresses although the mode of action is still elusive (Alcázar et al., 2011).

Unique features of Thellungiella metabolome were the very high levels of amino acids. Some amino acids are known to contribute to the tolerance to abiotic stresses. Proline is one of the well-documented osmoprotectants and some other amino acids including branched chain amino acids are accumulated under various stress conditions (Obata and Fernie, 2012). Interestingly, Thellungiella further accumulated proline and hydroxyproline during recovery from cold, which may be relevant for protection against subsequent or sustained low temperature stress. Proline is a well-known osmoprotectant that accumulates in response to drought or chilling in a number of species but Haberlea does not utilize this amino acid for protective purposes, as proline levels in Haberlea are low and, unlike Arabidopsis, do not elevate during chilling. These observations suggest that Arabidopsis and Thellungiella take advantage of amino acids and amino acid derivertives for stress adaptation in contrast to Haberlea, which accumulates sugars. The reason of this preference is unclear since the known functions of sugars and amino acids in stress tolerance are similar (Obata and Fernie, 2012). It may be due to the energetic and/or nutritional cost to produce large amount of metabolites belonging to these groups.

### Comparative analysis between low temperature- and dehydration-induced metabolic responses of *Haberlea rhodopensis*

The changes in metabolite levels of Haberlea during low temperature stress were compared with the recently conducted metabolome analysis of this species during drought stress and desiccation (Gechev et al., 2013a). Low temperature and drought are distinct abiotic stresses but both of them lead to loss of cellular water, hence specific as well as common metabolic responses are expected. The most obvious common response during both stresses was the massive accumulation of sucrose, which identifies this metabolite as a common protector against the two different types of osmotic shock in Haberlea. In both cases, sucrose accumulation is accompanied by elevated maltose levels, implying that starch degradation occurs during drought and low temperature stress—most probably as a carbon and energy source. During drought, this is accompanied by reduction of glucose and sucrose levels. During cold and especially upon recovery, however, the two sugars actually increase, especially upon recovery.

Apart from these few examples, most metabolites responded to the two stresses differently, suggesting very specific metabolome reconfigurations for drought and low temperature overall. Many key metabolites like trehalose, proline, citrate, succinate, and malate remained unchanged during low temperature, while all of them decreased during drought and desiccation. These metabolites, some of them well-known osmoprotectors and/or signaling molecules, may not be involved in acquiring drought tolerance but could well be part of the cold stress defence. Asparagine and aspartate levels decreased during drought/desiccation but increased during low temperature treatment. Aspartic acid was also induced by cold in *F. vesca* (Rohloff et al., 2012) and could contribute to the low temperature defence in *H. rhodopensis*. Proline and several organic acids related to the tricarboxylic acid cycle, including citrate, succinate, and malate, decreased during drought and desiccation but remained constant during low temperature stress. Trehalose levels also remained constant during chilling, while they dropped during dehydration and subsequent rehydration. GABA, a well-known stress signaling molecule and growth regulator, may on the other hand be involved primarily in the molecular mechanisms of desiccation tolerance, as its levels dramatically increase during desiccation but not during low temperature stress.

In conclusion, the three species have intrinsic differences in their metabolomes in the absence of stress and respond differently to chilling, implying unique strategies to counteract low temperature stress. Haberlea's high levels of galactinol, *myo*-inositol, sorbitol, and many sugars provide this species with steady-state metabolome which is configured to encounter the consequences of the low temperature stress already during the optimal growth conditions. While the oxidative stress-protective properties of galactinol and the osmoprotective as well as signaling properties of sucrose and trehalose are well-documented, the function of the other sugars such as fucose, rhamnose, and other monosaccharides in low temperature stress remains to be studied. Glucose and fructose, for example, may have dual functions: on one side, they are substrate for the increased sucrose synthesis, but on the other side they together with the rhamnose and fucose may play a role in the reconfiguration of the cell wall polysaccharides. Arabidopsis could utilize another defensive strategy based on transient cold-induced accumulation of sucrose, putrescine, and proline. It should also be noted that sugars in general may be involved in the cold acclimation rather than a specific sugar (Korn et al., 2008; Zuther et al., 2012). The high number of unidentified metabolites exclusively present in *H. rhodopensis* (Gechev et al., 2013a) suggests that there may be unique compounds this species synthesizes to protect itself from abiotic and oxidative stress. The accumulation of a large variety of metabolites is likely to contribute to the establishment of a robust system to cope with environmental stresses in Haberlea. Further study of the chemical identity of the unidentified metabolites may thus reveal new compounds with powerful stress protective functions (Gechev et al., 2013a). Thellungiella utilizes a third strategy, based on pre-adaptation using amino acids and polymines, transient cold-induced accumulation of putrescine and amino acids such as alanine and aspartate, and sustained accumulation of sucrose and proline. The latter may give Thellungiella an advantage relative to Arabidopsis in future stress encounters.

## Author contributions

Tsanko S. Gechev designed the research; Maria Benina, Toshihiro Obata, Nikolay Mehterov, Ivan Ivanov, Veselin Petrov, Valentina Toneva, Alisdair R. Fernie and Tsanko S. Gechev performed the experiments or/and analyzed the data; Maria Benina, Toshihiro Obata, Alisdair R. Fernie and Tsanko S. Gechev wrote the paper.

## Conflict of interest statement

The authors declare that the research was conducted in the absence of any commercial or financial relationships that could be construed as a potential conflict of interest.

## References

[B1] Alcázar R. Cuevas J. C. Planas J. Zarza X. Bortolotti C. Carrasco P. (2011). Integration of polyamines in the cold acclimation response. Plant Sci. 180, 31–38 10.1016/j.plantsci.2010.07.022 21421344

[B2] Apostolova E. Rashkova M. Anachkov N. Denev I. Toneva V. Minkov I. (2012). Molecular cloning and characterization of cDNAs of the superoxide dismutase gene family in the resurrection plant *Haberlea rhodopensis*. Plant Physiol. Biochem. 55, 85–92 10.1016/j.plaphy.2012.03.015 22562018

[B3] Battaglia M. Olvera-Carrillo Y. Garciarrubio A. Campos F. Covarrubias A. A. (2008). The enigmatic LEA proteins and other hydrophilins. Plant Physiol. 148, 6–24 10.1104/pp.108.120725 18772351 PMC2528095

[B4] Carillo P. Feil R. Gibon Y. Satoh-Nagasawa N. Jackson D. Bläsing O. E. (2013). A fluorometric assay for trehalose in the picomole range. Plant Methods 9, 21 10.1186/1746-4811-9-21 23786766 PMC3698175

[B5] Carvallo M. A. Pino M. T. Jeknic Z. Zou C. Doherty C. J. Shiu S. H. (2011). A comparison of the low temperature transcriptomes and CBF regulons of three plant species that differ in freezing tolerance: *Solanum commersonii*, *Solanum tuberosum*, and *Arabidopsis thaliana*. J. Exp. Bot. 62, 3807–3819 10.1093/jxb/err066 21511909 PMC3134341

[B6] Cook D. Fowler S. Fiehn O. Thomashow M. F. (2004). A prominent role for the CBF cold response pathway in configuring the low-temperature metabolome of Arabidopsis. Proc. Natl. Acad. Sci. U.S.A. 101, 15243–15248 10.1073/pnas.0406069101 15383661 PMC524070

[B7] Crowe J. H. Hoekstra F. A. Crowe L. M. (1992). Anhydrobiosis. Annu. Rev. Physiol. 54, 579–599 10.1146/annurev.ph.54.030192.003051 1562184

[B8] Dauwe R. Holliday J. A. Aitken S. N. Mansfield S. D. (2012). Metabolic dynamics during autumn cold acclimation within and among populations of Sitka spruce (*Picea sitchensis*). New Phytol. 194, 192–205 10.1111/j.1469-8137.2011.04027.x 22248127

[B9] Davik J. Koehler G. From B. Torp T. Rohloff J. Eidem P. (2013). Dehydrin, alcohol dehydrogenase, and central metabolite levels are associated with cold tolerance in diploid strawberry (*Fragaria* spp.). Planta 237, 265–277 10.1007/s00425-012-1771-2 23014928

[B10] Dinakar C. Djilianov D. Bartels D. (2012). Photosynthesis in desiccation tolerant plants: energy metabolism and antioxidative stress defense. Plant Sci. 141, 436–445 10.1016/j.plantsci.2011.01.018 22118613

[B11] Drennan P. M. Smith M. T. Goldsworthy D. Van Staden J. (1993). The occurrence of trehalose in the leaves of the desiccation-tolerant angiosperm *Myrothmannus flabellifolius* Welw. J. Plant Physiol. 142, 493–496 10.1016/S0176-1617(11)81257-5

[B12] Farrant J. Brandt W. Lindsey G. G. (2007). An overview of mechanisms of desiccation tolerance in selected angiosperm resurrection plants. Plant Stress J. 1, 72–84

[B13] Fernie A. R. Aharoni A. Willmitzer L. Stitt M. Tohge T. Kopka J. (2011). Recommendations for reporting metabolite data. Plant Cell 23, 2477–2482 10.1105/tpc.111.086272 21771932 PMC3226225

[B14] Gechev T. Benina M. Obata T. Tohge T. Sujeeth N. Minkov I. (2013a). Molecular mechanisms of desiccation tolerance in the resurrection glacial relic *Haberlea rhodopensis*. Cell. Mol. Life Sci. 70, 689–709 10.1007/s00018-012-1155-6 22996258 PMC11113823

[B15] Gechev T. Mehterov N. Denev I. Hille J. (2013b). A simple and powerful approach for isolation of Arabidopsis mutants with increased tolerance to H_2_O_2_-induced cell death. Methods Enzymol. 527, 203–220 10.1016/B978-0-12-405882-8.00011-8 23830633

[B16] Gechev T. Dinakar C. Benina M. Toneva V. Bartels D. (2012). Molecular mechanisms of desiccation tolerance in resurrection plants. Cell. Mol. Life Sci. 69, 3175–86 10.1007/s00018-012-1088-0 22833170 PMC11114980

[B17] Georgieva T. Christov N. Djilianov D. (2012). Identification of desiccation-regulated genes by cDNA-AFLP in *Haberlea rhodopensis*: a resurrection plant. Acta Physiol. Plant. 34, 1055–1066 10.1007/s11738-011-0902-x

[B18] Gong Q. Li P. Ma S. Indu Rupassara S. Bohnert H. J. (2005). Salinity stress adaptation competence in the extremophile *Thellungiella halophila* in comparison with its relative *Arabidopsis thaliana*. Plant J. 44, 826–839 10.1111/j.1365-313X.2005.02587.x 16297073

[B18a] Hannah M. A. Wiese D. Freund S. Fiehn O. Heyer A. G. Hincha D. K. (2006). Natural genetic variation of freezing tolerance in Arabidopsis. Plant Physiol. 142, 98–112 10.1104/pp.106.081141 16844837 PMC1557609

[B19] Ingram J. Bartels D. (1996). The molecular basis of dehydration tolerance in plants. Annu. Rev. Plant Physiol. Plant Mol. Biol. 47, 377–403 10.1146/annurev.arplant.47.1.377 15012294

[B21] Kirch H. H. Nair A. Bartels D. (2001). Novel ABA- and dehydration-inducible aldehyde dehydrogenase genes isolated from the resurrection plant *Craterostigma plantagineum* and *Arabidopsis thaliana*. Plant J. 28, 555–567 10.1046/j.1365-313X.2001.01176.x 11849595

[B22] Kopka J. Schauer N. Krueger S. Birkemeyer C. Usadel B. Bergmuller E. (2005). GMD@CSB.DB: the Golm Metabolome Database. Bioinformatics 21, 1635–1638 10.1093/bioinformatics/bti236 15613389

[B23] Korn M. Peterek S. Mock H. P. Heyer A. G. Hincha D. K. (2008). Heterosis in the freezing tolerance, and sugar and flavonoid contents of crosses between *Arabidopsis thaliana* accessions of widely varying freezing tolerance. Plant Cell Environ. 31, 813–827 10.1111/j.1365-3040.2008.01800.x 18284584 PMC2440548

[B24] Lee Y. P. Babakov A. de Boer B. Zuther E. Hincha D. K. (2012). Comparison of freezing tolerance, compatible solutes and polyamines in geographically diverse collections of *Thellungiella* sp. *and Arabidopsis thaliana accessions.* BMC Plant Biol. 12:131 10.1186/1471-2229-12-131 22863402 PMC3464606

[B25] Lisec J. Schauer N. Kopka J. Willmitzer L. Fernie A. R. (2006). Gas chromatography mass spectrometry-based metabolite profiling in plants. Nat. Protoc. 1, 387–396 10.1038/nprot.2006.59 17406261

[B26] Luedemann A. Strassburg K. Erban A. Kopka J. (2008). TagFinder for the quantitative analysis of gas chromatography-mass spectrometry (GC–MS)-based metabolite profiling experiments. Bioinformatics 24, 732–737 10.1093/bioinformatics/btn023 18204057

[B27] Lunn J. E. Feil R. Hendriks J. H. M. Gibon Y. Morcuende R. Osuna D. (2006). Sugar-induced increases in trehalose 6-phosphate are correlated with redox activation of ADP glucose pyrophosphorylase and higher rates of starch synthesis in *Arabidopsis thaliana.* Biochem. J. 397, 139–148 10.1042/BJ20060083 16551270 PMC1479759

[B28] Martinelli T. (2008). *In situ* localization of glucose and sucrose in dehydrating leaves of *Sporobolus stapfianus*. J. Plant Physiol. 165, 580–587 10.1016/j.jplph.2007.01.019 17765358

[B29] Mehterov N. Balazadeh S. Hille J. Toneva V. Mueller-Roeber B. Gechev T. (2012). Oxidative stress provokes distinct transcriptional responses in the stress-tolerant *atr7* and stress-sensitive *loh2 Arabidopsis thaliana* mutants as revealed by multi-parallel quantitative real-time PCR analysis of ROS marker and antioxidant genes. Plant Physiol. Biochem. 59, 20–9 10.1016/j.plaphy.2012.05.024 22710144

[B30] Moore J. P. Nguema-Ona E. E. Vicré-Gibouin M. Sørensen I. Willats W. G. Driouich A. (2012). Arabinose-rich polymers as an evolutionary strategy to plasticize resurrection plant cell walls against desiccation. Planta 237, 739–754 10.1007/s00425-012-1785-9 23117392

[B31] Mowla S. B. Thomson J. A. Farrant J. M. Mundree S. G. (2002). A novel stress-inducible antioxidant enzyme identified from the resurrection plant *Xerophyta viscosa* Baker. Planta 215, 716–726 10.1007/s00425-002-0819-0 12244436

[B32] Nishizawa A. Yabuta Y. Shigeoka S. (2008). Galactinol and raffinose constitute a novel function to protect plants from oxidative damage. Plant Physiol. 147, 1251–1263 10.1104/pp.108.122465 18502973 PMC2442551

[B33] Norwood M. Toldi O. Richter A. Scott P. (2003). Investigation into the ability of roots of the poikilohydric plant *Craterostigma plantagineum* to survive dehydration stress. J. Exp. Bot. 54, 2313–2321 10.1093/jxb/erg255 12947051

[B34] Norwood M. Truesdale M. R. Richter A. Scott P. (2000). Photosynthetic carbohydrate metabolism in the resurrection plant *Craterostigma plantagineum*. J. Exp. Bot. 51, 159–165 10.1093/jexbot/51.343.159 10938822

[B35] Obata T. Fernie A. R. (2012). The use of metabolomics to dissect plant responses to abiotic stresses. Cell. Mol. Life Sci. 69, 3225–3243 10.1007/s00018-012-1091-5 22885821 PMC3437017

[B36] Oliver M. J. Guo L. Alexander D. C. Ryals J. A. Wone B. W. Cushman J. C. (2011). A sister group contrast using untargeted global metabolomic analysis delineates the biochemical regulation underlying desiccation tolerance in *Sporobolus stapfianus.* Plant Cell 23, 1231–1248 10.1105/tpc.110.082800 21467579 PMC3101564

[B39] Rodriguez M. C. Edsgärd D. Hussain S. S. Alquezar D. Rasmussen M. Gilbert T. Mundy J. (2010). Transcriptomes of the desiccation-tolerant resurrection plant *Craterostigma plantagineum*. Plant J. 63, 212–228 10.1111/j.1365-313X.2010.04243.x 20444235

[B40] Rohloff J. Kopka J. Erban A. Winge P. Wilson R. C. Bones A. M. (2012). Metabolite profiling reveals novel multi-level cold responses in the diploid model *Fragaria vesca* (woodland strawberry). Phytochemistry 77, 99–109 10.1016/j.phytochem.2012.01.024 22370221

[B41] Sanchez D. H. Pieckenstain F. L. Escaray F. Erban A. Kraemer U. Udvardi M. K. (2011). Comparative ionomics and metabolomics in extremophile and glycophytic Lotus species under salt stress challenge the metabolic pre-adaptation hypothesis. Plant Cell Environ. 34, 605–617 10.1111/j.1365-3040.2010.02266.x 21251019

[B42] Schluepmann H. Pellny T. van Dijken A. Smeekens S. Paul M. (2003). Trehalose 6-phosphate is indispensable for carbohydrate utilization and growth in *Arabidopsis thaliana*. Proc. Natl. Acad. Sci. U.S.A. 100, 6849–6854 10.1073/pnas.1132018100 12748379 PMC164535

[B43] Schmittgen T. D. Livak K. J. (2008). Analyzing real-time PCR data by the comparative Ct method. Nat. Protoc. 3, 1101–1108 10.1038/nprot.2008.73 18546601

[B44] Smeekens S. Ma J. Hanson J. Rolland F. (2010). Sugar signals and molecular networks controlling plant growth. Curr. Opin. Plant Biol. 13, 274–279 10.1016/j.pbi.2009.12.002 20056477

[B45a] Thomashow M. F. (2010). Molecular basis of plant cold acclimation: insights gained from studying the CBF cold response pathway. Plant Physiol. 154, 571–577 10.1104/pp.110.161794 20921187 PMC2948992

[B46] Van Den Dries N. Facchinelli F. Giarola V. Phillips J. R. Bartels D. (2011). Comparative analysis of LEA-like 11-24 gene expression and regulation in related plant species within the Linderniaceae that differ in desiccation tolerance. New Phytol. 190, 75–88 10.1111/j.1469-8137.2010.03595.x 21231934

[B47] Xia J. Mandal R. Sinelnikov I. V. Broadhurst D. Wishart D. S. (2012). MetaboAnalyst 2.0-a comprehensive server for metabolomic data analysis. Nucleic Acids Res. 40, W127–W133 10.1093/nar/gks374 22553367 PMC3394314

[B48] Yobi A. Wone B. W. Xu W. Alexander D. C. Guo L. Ryals J. A. (2012). Comparative metabolic profiling between desiccation-sensitive and desiccation-tolerant species of Selaginella reveals insights into the resurrection trait. Plant J. 72, 983–999 10.1111/tpj.12008 23061970

[B49] Yobi A. Wone B. W. Xu W. Alexander D. C. Guo L. Ryals J. A. (2013). Metabolomic profiling in *Selaginella lepidophylla* at various hydration states provides new insights into the mechanistic basis of desiccation tolerance. Mol. Plant 6, 369–385 10.1093/mp/sss155 23239830

[B50] Zhang Y. Butelli E. De Stefano R. Schoonbeek H. J. Magusin A. Pagliarani C. (2013). Anthocyanins double the shelf life of tomatoes by delaying overripening and reducing susceptibility to gray mold. Curr. Biol. 23, 1094–1100 10.1016/j.cub.2013.04.072 23707429 PMC3688073

[B51] Zou C. Sun K. Mackaluso J. D. Seddon A. E. Jin R. Thomashow M. F. (2011). Cis-regulatory code of stress-responsive transcription in *Arabidopsis thaliana*. Proc. Natl. Acad. Sci. U.S.A. 108, 14992–14997 10.1073/pnas.1103202108 21849619 PMC3169165

[B52] Zuther E. Schulz E. Childs L. H. Hincha D. K. (2012). Clinal variation in the non-acclimated and cold-acclimated freezing tolerance of *Arabidopsis thaliana* accessions. Plant Cell Environ. 35, 1860–1878 10.1111/j.1365-3040.2012.02522.x 22512351

